# Assessment of Consistency between Claims and References Referred to in Pharmaceutical Advertising Brochures in the Kingdom of Saudi Arabia

**DOI:** 10.7759/cureus.3907

**Published:** 2019-01-17

**Authors:** Tareq M Al-Tuhaifi, Ayman M Awad, Ahmed Abu-Zaid, Abdulaziz M Eshaq, Najwa Mohammad, Sandrella I Zebian, Abdulkarim G Sulaihim, Yara Alburaidi, Ahmed Fothan, Omer Kaweilh, Dileep K Rohra, Abdulhadi A Alamodi

**Affiliations:** 1 Pathology, Alfaisal University College of Medicine, Riyadh, SAU; 2 Ophthalmology, Alfaisal University College of Medicine, Riyadh, SAU; 3 Oncology, Alfaisal University College of Medicine, Riyadh, SAU; 4 Internal Medicine, Alfaisal University College of Medicine, Riyadh, USA; 5 Internal Medicine, Alfaisal University College of Medicine, Riyadh, SAU; 6 Family Medicine, Alfaisal University College of Medicine, Riyadh, SAU; 7 Surgery, Riyadh Elm University, Riyadh, SAU; 8 Dentistry, Riyadh Elm University, Riyadh, SAU; 9 Miscellaneous, Alfaisal University College of Medicine, Riyadh, SAU; 10 Pharmacology, Alfaisal University College of Medicine, Riyadh, SAU; 11 Surgery, Alfaisal University College of Medicine, Riyadh, SAU

**Keywords:** drug advertisement brochures, pharmaceutical, references, quality of evidence

## Abstract

Drug advertisement brochures (DABs) contain claims that are often supplemented by references in medical literature. Several studies have evaluated the DABs as they are commonly distributed by drug companies to practicing physicians. The objective of this study is to assess the consistency between the claims and references referred to in the DABs in Saudi Arabia. DABs were collected from medical practitioners in Riyadh, Saudi Arabia. Authors developed a protocol to be followed for quality assessment of the DABs. The vast majority of cited scientific papers were indexed in PubMed. Consequently, each reference was categorized as: justifiable, false, exaggerated or ambiguous. A total of 89 DABs were collected; 48 (53.9%) brochures were excluded from further analysis and the remaining 41 brochures (46.1%) contained 240 references with an approximate average of 5.9 references per DAB. A total of 201 cited papers were traced (83.8%). The majority of references (93.0%) supported the claims for which they were cited. However, 1.5%, 4.0% and 1.5% of claims were deemed inaccurate/false, exaggerated, and ambiguous, respectively. This study supports that the majority of the claims made in the DABs of pharmaceutical companies in Saudi Arabia were unreferenced. However, most of the evidence presented to substantiate claims made was considered true.

## Introduction

Drug companies have adopted several ways to promote products targeted to health care professionals. Pharmaceutical companies in Saudi Arabia, like those in many other countries, mainly advertise their products by direct approach to medical practitioners and distribution of advertisement brochures, pamphlets, and samples [1]. The drugs are promoted through a series of claims stating a drug’s indication, effectiveness, cost-effectiveness, prices, and its possible side effects. These claims should always be supplemented by references that prove their accuracy [2]. The Saudi Ministry of Health (SMOH) guarantees that available information on the drug promotional material is not inconsistent with the labeling on the drugs [3]. The accuracy of the information conveyed through claims on drug advertisements has not yet been analyzed in Saudi Arabia. In other countries, however, drug advertisement flyers have been thoroughly appraised and censured for their inaccuracies, manipulative effects on physicians’ prescribing behavior [4-6] and ethical issues [7-9].

In order to validate the promotional claims, advertisements frequently cite studies that have been published in different journals that may impress health care professionals and/or influence their decision in prescribing the promoted medication to their patients [8]. The efficacy of these references — as means for critical assessment of the drugs’ claims — remains irrefutable despite the fact that studies have shown that most of the references cited on these brochures lacked sufficient data to factually prove these claims [10-13].

In a previous study in Pakistan, it was shown that pharmaceutical advertisement agents and their brochures are the main conveyor of recently developed drugs to physicians [14]. Therefore, ideally, the information provided on the advertisements and its supporting evidence should be irrefutable in order to support physicians in practicing evidence-based medicine. It was also reported that the claims made by pharmaceutical companies in their promotional material [13] and the references substantiating these claims are inaccurate in Pakistan [14-16]. Thus, considering the serious influence these advertisements have on the prescribing behavior of physicians [16,17], this study was designed to assess the consistency between the evidence (references) and claims presented in advertising brochures in the Kingdom of Saudi Arabia.

## Materials and methods

Brochures were collected, by first five authors, from physicians working in private and governmental hospitals in Riyadh, Saudi Arabia, from July 1 to 31, 2012. A sample of 89 flyers was obtained. All information in each brochure was extracted by the first five authors. From this set of collected brochures, two types were excluded and were not subjected to any further analysis. The first type included those brochures that did not cite any references whereas the second type included those brochures in which reference(s) was/were cited but were not paired with any particular claim.

Investigators then followed a strategy for assessment and analysis of the citations attached within the remaining advertisement materials. Using the infrastructure of Alfaisal University, the references were categorized by source and were then traced in available databases including PubMed and the Internet to ensure their accessibility within indexed journals. All authors were engaged in this step except the last two authors. In case full-text published articles were not available, investigators evaluated their related abstracts on PubMed. After this extensive investigation, references were finally divided into two main categories; ‘traceable’ and ‘non-traceable’. A reference was adjudged as ‘non-traceable’ if it was unavailable in literature or was inaccessible due to incomplete bibliographic reference requirements such as the author’s name, journal’s title, year of publication, issue, page numbers, volume or supplement. Misspelled information such as the journal’s title or author’s name had been rarely detected and were ignored by authors when automatically corrected by spelling check tool of the database as in PubMed. Eventually, the authors were divided into two teams of three individuals in each team. Each team reviewed the claims mentioned in the brochures and assessed to what extent the reference supported each claim according to the following categories:

Justifiable: a claim was considered ‘justifiable’ when it sufficiently was supported by its reference.

False: a claim was labeled as ‘false or inaccurate’ when the reference did not match the considered claim.

Exaggerated: a claim was considered ‘exaggerated’ when referring to unnecessary application/information in the literature that had been inadequately augmented by a claim.

Ambiguous: a claim was regarded as ‘ambiguous’ when there was no clear relation between a reference’s evidence and a designated claim which was difficult to be interpreted by investigators. Any conflict between the two teams of authors was resolved by the last two authors.

## Results

A total of 89 different brochures were collected. Forty-eight (53.9%) brochures were excluded from the study according to the exclusion criteria mentioned in the Methods section. Briefly, 39 brochures did not cite any references and nine did not pair their references with their claims. The remaining 41 brochures were considered for further analysis (Poster: Altuhaifi T, AlAmodi AA, Zebian S, Rohra D. Critical Assessment of References Provided in Pharmaceutical Advertising Brochures in the Kingdom of Saudi Arabia. Undergraduate Research Conference; 2016).

These 41 brochures contained a total of 240 references with an average of 5.9 references per brochure. A breakdown of the references’ sources is depicted in Table 1. The references classified under ‘other’ are those for which no single class definition could be determined. These references quoted posters, magazines and various companies’ reports on products. As depicted in Table 1, 201 citations (83.8%) were traced and analyzed on various databases. However, the remaining 39 (16.2%) citations could not be retrieved due to various reasons including unavailability of the mentioned sources, untraceable referenced books, non-indexed journals or data on file reports, and misspelled data. Each traceable reference (n = 201) was further evaluated as to whether it supported the claim for which it was cited or not. The majority of the references (n = 187; 93.0%) supported the claims for which they were cited (justifiable). However, three (1.5%), eight (4.0%) and three (1.5%) claims were adjudged as inaccurate/false, exaggerated and ambiguous, respectively. Around 34 (82.93%) brochures had all justifiable references, whereas seven (17.07%) brochures had a mix of justifiable and non-justifiable references. There were no brochures that included only non-justifiable references.

**Table 1:** Distribution of sources of citations. WHO: World Health Organization

Source	Total citations %	Traceable n %	Non-traceable n %
Pubmed-indexed journals	73.7	72.9	0.8
Non-Pubmed-indexed journals	11.7	5.4	6.3
Reference books	2.9	0	2.9
Online addresses	2.9	1.7	1.2
WHO/National Health Guidelines	2.9	2.1	0.8
Product manual	2.1	0.8	1.3
Others (reports, magazines)	3	0.8	2.9
Total	100	83.8	16.2

## Discussion

Drug promotion by pharmaceutical companies through the direct approach of health care professionals continues to be the dominant marketing strategy. It has been shown that the physicians’ prescribing behavior is influenced by these advertisements [17]. Consequently, the acceptance of drug’s promotional claims without any questioning can contribute to irrational prescribing. Therefore, the aim of the study was to evaluate the consistency between the evidence presented and the claims in the pharmaceutical advertisement brochures. This aim was achieved by tackling two questions. First, whether the claims which prompt the prescribers are validated by evidence or not; second, whether the evidence presented to support the claim is justifiable or not (Table 2).

**Table 2:** Various examples of false, exaggerated and ambiguous claims in drug advertisements with their evidence statements. IU: International Units; HER2: Human Epidermal Growth Factor Receptor 2; PTH: Parathyroid Hormone.

#	Drug	Pharmaco-logical class	Claim	Evidence statement	Remark
1	Calcium carbonate + Cholecalciferol	Calcium and vitamin D supplement	At least 800 IU/day of vitamin D is needed for maximum suppression of PTH, maximum absorption of calcium, and has been shown to prevent fractures in older adults.	Partially true, but there was no evidence from the article about the relation with the maximum suppression of PTH and the absorption of calcium from the gut.	Exaggerated
2	Trastuzumab	HER2/neu receptor monoclonal antibody	It rebuilds hope.	It is a very general statement. Three articles were cited to substantiate this claim but none of them mentioned that it can rebuild hope.	Ambiguous
3	Cefaclor	2^nd^ generation cephalosporin	Curative rate 100% in pneumonia.	The overall satisfactory clinical response was 97.3% for azithromycin patients and 100% for cefaclor patients. However, the clinical cure rates of azithromycin and cefaclor were 46.9% and 41.0%, respectively.	False
4	Capecitabine	Anticancer drug	Tried and trusted for 1,500,000 patients	The evidence mentioned in the report that this medication had been tried and trusted for 0.5 million patients.	Exaggerated

As far as the first question is concerned, it was noted that more than half of the brochures did not contain any evidence/reference. Either these brochures did not contain any reference, or, they contained a cluster of claims and references with no indication of which claim is paired/supported by which reference. This effectively prevented further analysis of the evidence, consequently excluding those brochures from the study. However, we noted that the majority of excluded brochures were related to medical conditions such as allergic diseases, skin diseases, and vitamins’ supplements. Lankinen et al. also reported that even medical journals’ advertisements published had only 38% of the claims supported by references, while the rest of the claims were unreferenced [18].

Regarding the second question, whether the claim is justifiable or not in the light of cited reference, it was revealed that an overwhelming majority of the claims (93%) were made with the evidence presented. This is quite remarkable when the collected data is compared with previously published reports. These studies have reported a large percentage of claims which could not be considered as justifiable [11-13,18]. Inappropriate use of references in journal advertising led the authors of these studies to conclude that the availability of references does not always guarantee the quality of claims. We speculate that there are several reasons to why such discrepancy in our data compared to previous reports. First, studies by Kasper et al. and Wilkes et al. were published early in the nineties. Secondly, different countries have different regulations in terms of drug promotional distribution, and thirdly, the samples chosen for analysis may contribute to the differences between different reports. Gutknecht found that out of 187 distinct drug advertisements, only 33 were evidence-based ones. In contrast, in our study, almost half of the collected brochures were with no supporting evidence supporting the claims. However, the strength of Gutknecht study is that a quantitative critical analysis was applied by evaluating measure that helps in the decision process of a physician such as absolute values, blinding, randomization and intention to treat numbers. Our study, on the other hand, followed a qualitative approach by evaluating whether the claims are supported by evidence and if so to what extent the claims being presented match the supporting evidence and if they are true, false, ambiguous or exaggerated.

There are a few limitations to this study. One is the sample size and sample technique. A total number of 89 brochures is not representative. Second, brochures were collected in 2012 during one month from only one big city and the data may be different if the printed promotional materials distributed in other cities and smaller towns were targeted. It is possible that the trend over the span of the past five years might have changed and further large-scale study with more comprehensive quantitative analysis is required to rectify this dilemma. Another limitation of the study was that no attempt was made to define what was meant by the quality of evidence. No research was done to validate the authenticity of the claims or refute them on the basis of available evidence. Rather than analyzing the type of study, journal, and so on, the definition was based on the presence or the absence of evidence and the extrapolation of the findings of the studies which were cited in the printed material by the pharmaceutical companies.

## Conclusions

Our study shows that in Saudi Arabia approximately half of the drug advertisement brochures (DABs) and their claims were presented without any evidence. However, the majority of evidence presented in the other half of the DABs was accurate.
